# Complete Genome Sequence of Lleida Bat Lyssavirus

**DOI:** 10.1128/genomeA.01427-16

**Published:** 2017-01-12

**Authors:** Denise A. Marston, Richard J. Ellis, Emma L. Wise, Nidia Aréchiga-Ceballos, Conrad M. Freuling, Ashley C. Banyard, Lorraine M. McElhinney, Xavier de Lamballerie, Thomas Müller, Anthony R. Fooks, Juan E. Echevarría

**Affiliations:** a Wildlife Zoonoses & Vector-Borne Diseases Research Group, Animal and Plant Health Agency (APHA), Surrey, United Kingdom; b Surveillance and Laboratory Services Department, APHA, Surrey, United Kingdom; c UMR_D 190, Emergence de Pathologies Virales, Aix Marseille University, Marseille, France; d Instituto de Salud Carlos III, Madrid, Spain; e Laboratorio de Rabia, Instituto de Diagnóstico y Referencia Epidemiológicos, Ciudad de México, Mexico; f Friedrich Loeffler Institute (FLI), Institute of Molecular Virology and Cell Biology, Greifswald-Insel Riems, Germany; g Institute of Infection and Global Health, University of Liverpool, Liverpool, United Kingdom; h Institute for Infection and Immunity, St. George’s Hospital Medical School, University of London, London, United Kingdom; i Ciber de Epidemiología y Salud Pública (CIBERESP), Madrid, Spain

## Abstract

All lyssaviruses (family *Rhabdoviridae*) cause the disease rabies, an acute progressive encephalitis for which, once symptoms occur, there is no effective cure. Using next-generation sequencing, the full-genome sequence for a novel lyssavirus, Lleida bat lyssavirus (LLEBV), from the original brain of a common bent-winged bat has been confirmed.

## GENOME ANNOUNCEMENT

The lyssavirus genome consists of a single-stranded negative-sense RNA of approximately 12 kb. The International Committee for the Taxonomy of Viruses recognizes 14 distinct lyssavirus species, with Lleida bat lyssavirus (LLEBV) and Gannoruwa bat lyssavirus (GBLV) awaiting classification (1, 2).

LLEBV was detected in a common bent-winged bat (*Miniopterus schreibersii*) found in Lleida, Spain, during July 2011 (1). Initial analysis of the partial nucleoprotein (N) sequence confirmed that this virus was most closely related to members of the *Lyssavirus* genus, grouping with West Caucasian bat lyssavirus and Ikoma lyssavirus sequences. Full-genome sequencing of the original brain material was undertaken to genetically characterize this virus. Generation of the full-genome sequence was particularly significant, as initial attempts at virus isolation failed. Viral RNA was extracted using TRIzol, and host genomic DNA and rRNA were depleted as described previously (3). Double-stranded cDNA (ds-cDNA) was synthesized using random hexamers and a cDNA synthesis system (Roche). The ds-cDNA was purified using AMPure XP magnetic beads (Beckman Coulter, Inc.), and 1 ng was used with the Nextera XT DNA sample preparation kit (Illumina), according to the manufacturer’s instructions (omitting the bead normalization step) and sequenced using an Illumina MiSeq with 2 × 150-bp paired-end reads.

Mapping the sequencing data (6,651,896 reads) to the most closely related lyssavirus genome (Ikoma lyssavirus [IKOV]) failed to generate a consensus sequence. Therefore, the reads were processed to remove host genome by mapping to a mouse reference (sufficient homology to bat) using BWA version 0.7.5a-r405 (4). The remaining unmapped reads were assembled using IVA (5). Two virus-specific contiguous sequences (contigs) were obtained, which were confirmed by mapping the original data with BWA and consensus calling with a modified SAMtools script (6). The total number of assembled viral reads was 3,610 (0.25% of the host-depleted reads). The two contigs covered all coding regions, but a section of the G-L region was absent, and the genomic ends were atypical lengths. The G-L intergenic region and genomic ends were confirmed by Sanger sequencing of PCR amplicons, obtained using primers designed from the next-generation sequencing (NGS) consensus sequences, and paired with conserved primers that bind the conserved lyssavirus genomic termini (7). The sequence obtained by combining the two NGS contigs and three PCR amplicon sequences was used to map the original reads and determine the consensus sequence as described above. The total number of assembled viral reads for LLEBV was 12,629 (0.19% of total reads). The entire genome, apart from the last two nucleotides, was covered by NGS reads.

The genetic organization of the LLEBV genome is similar to that of other lyssaviruses, with a complete genome size of 11,931 nucleotides (nt). The open reading frame (ORF) lengths are as follows: 3′ untranslated region (UTR), 70 nt; N-ORF, 1,353 nt; N-P intergenic region, 68 nt; P-ORF, 870 nt; P-M intergenic region, 74 nt; M-ORF, 609 nt; M-G intergenic region, 198 nt; G-ORF, 1,578 nt; G-L intergenic region, 608 nt; L-ORF, 6,381 nt; and 5′ UTR, 122 nt. These data will contribute to our understanding of lyssavirus diversity and evolution and further our knowledge of vaccine-induced immunity and protection.

### Accession number(s).

The complete genomic sequence of LLEBV has been deposited in GenBank under accession number KY006983.
